# Morphological Characterization of *Mycobacterium tuberculosis* in a MODS Culture for an Automatic Diagnostics through Pattern Recognition

**DOI:** 10.1371/journal.pone.0082809

**Published:** 2013-12-16

**Authors:** Alicia Alva, Fredy Aquino, Robert H. Gilman, Carlos Olivares, David Requena, Andrés H. Gutiérrez, Luz Caviedes, Jorge Coronel, Sandra Larson, Patricia Sheen, David A. J. Moore, Mirko Zimic

**Affiliations:** 1 Laboratorio de Bioinformática y Biología Molecular, Laboratorios de Investigación y Desarrollo, Facultad de Ciencias y Filosofía, Universidad Peruana Cayetano Heredia, Lima, Peru; 2 Laboratorio de Tuberculosis, Laboratorios de Investigación y Desarrollo, Facultad de Ciencias y Filosofía, Universidad Peruana Cayetano Heredia, Lima, Peru; 3 Department of International Health, School of Public Health, Johns Hopkins University, Baltimore, Maryland, United States of America; 4 College of Osteopathic Medicine, Michigan State University, East Lansing, Michigan, United States of America; 5 Department of Infectious Diseases and Immunity, Wellcome Centre for Clinical Tropical Medicine, Imperial College London, London, United Kingdom; California Department of Public Health, United States of America

## Abstract

Tuberculosis control efforts are hampered by a mismatch in diagnostic technology: modern optimal diagnostic tests are least available in poor areas where they are needed most. Lack of adequate early diagnostics and MDR detection is a critical problem in control efforts.

The Microscopic Observation Drug Susceptibility (MODS) assay uses visual recognition of cording patterns from *Mycobacterium tuberculosis* (MTB) to diagnose tuberculosis infection and drug susceptibility directly from a sputum sample in 7–10 days with a low cost.

An important limitation that laboratories in the developing world face in MODS implementation is the presence of permanent technical staff with expertise in reading MODS.

We developed a pattern recognition algorithm to automatically interpret MODS results from digital images. The algorithm using image processing, feature extraction and pattern recognition determined geometrical and illumination features used in an object-model and a photo-model to classify TB-positive images. 765 MODS digital photos were processed. The single-object model identified MTB (96.9% sensitivity and 96.3% specificity) and was able to discriminate non-tuberculous mycobacteria with a high specificity (97.1% *M. avium*, 99.1% *M. chelonae*, and 93.8% *M. kansasii)*. The photo model identified TB-positive samples with 99.1% sensitivity and 99.7% specificity.

This algorithm is a valuable tool that will enable automatic remote diagnosis using Internet or cellphone telephony. The use of this algorithm and its further implementation in a telediagnostics platform will contribute to both faster TB detection and MDR TB determination leading to an earlier initiation of appropriate treatment.

## Introduction

Tuberculosis (TB) is a contagious airborne disease caused by the bacteria *Mycobacterium tuberculosis* (MTB). TB is widespread and deadly, and causes the highest number of deaths worldwide. An estimated of 8.7 million of new cases of TB and 1.4 million deaths occurs per year [1]. One third of the global population has latent TB infection, meaning that they are infected with *M. tuberculosis* but are not sick with the disease. Five to ten percent of infected people will eventually develop active TB disease in their lifetime [2].

In recent years, drug-resistant TB has emerged, largely due to delays in treatment, gaps in treatment protocol, and ineffective or delayed drug-susceptibility testing [3]. Multi-drug resistant tuberculosis (MDR-TB) is defined by the resistance of the bacillus to the most powerful TB drugs, isoniazid and rifampin, while extensively drug-resistant TB (XDR-TB) is also resistant to some second line drugs [4], [5]. World Health Organization estimated in the Global Tuberculosis Report 2012 that 3.7% (2.1–5.2%) of new cases of TB and 20% (13–26%) of previously treated cases have MDR-TB. XDR-TB was reported by 84 countries, and the proportion of MDR-TB cases with XDR-TB was approximately 9.0% [1]. In 2011 the World Health Organization endorsed a new phenotypic technique for TB diagnosis and drug susceptibility testing, Microscopic Observation of Drug Susceptibility (MODS), which is based on visual identification of a microscopic cording pattern characteristic of *M. tuberculosis* colonies during growth in a liquid phase [6], [7]. MODS use sputum samples to detect the presence of tuberculosis and MDR-TB accurately and with high sensitivity and specificity. The average time to detection of TB and MDR-TB is seven days [8].

A typical TB cord in a positive MODS culture, exhibits certain morphological and illumination characteristics. A TB positive cord has a particular length and width, and its shape is usually sinuous with a smooth border. Given that a TB cord has a circular transversal section, the light that passes through the diameter shows a high-transmitted brightness, while the light that passes through the border, gets refracted resulting in a lower brightness.

Despite the advantages of MODS and its potential to intensify and accelerate the global fight against TB, it still requires staff specifically trained to read the culture plates, which do not yet exist in remote laboratories lacking financial and technical resources, in the settings where most TB occurs. It is therefore important to improve TB and MDR TB detection using alternate sensitive approaches [9].

Because MODS involves the visual diagnosis of MTB, we hypothesized that computerized pattern recognition on digital images of MODS cultures could be used in the automatic detection of MTB growth.

Several studies on automated diagnostics of tuberculosis using pattern recognition yielded good results. Previous works analyzed sputum smears stained with auramine. Images were obtained by fluorescent microscopy and different algorithms and classifiers were used, including neural networks [10] and support vector machines, Gaussian mixture models and Bayesian decision theory [11]. These diagnostic methods reached up to 100% sensitivity and sensitivity per sample. Other studies analyzed sputum smear with Ziehl-Neelsen staining microscopy. Detection algorithms used Bayesian segmentation and Gaussian classifiers [12], [13], obtaining sensitivities and specificities above 95% for the classification of TB positive smears. All of these studies showed that pattern recognition has been applied successfully in the diagnosis of TB. However, to date there is no automated recognition for use with MODS culture.

Here we describe a method for automating MODS diagnosis using computer vision algorithm based on available techniques to the problem of detecting tuberculosis and MDR from MODS digital microscopy images. This computer algorithm will contribute with the global efforts to fight against tuberculosis.

## Materials and Methods

### Microscopic observation drug susceptibility (MODS) assay

**Figure 1:**
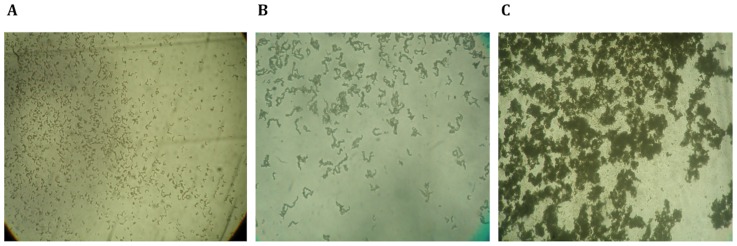
Temporal variation of the morphological characteristics of MTB cords in MODS culture observed at 100X total magnification. (A) Day 3 of culture; (B) Day 10 of culture; (C) Day 15 of culture. Cords observed at day 10 are highly specific for MTB.

The microscopic observation drug susceptibility (MODS) assay is a method based on microscopic examination of liquid media to detect characteristic cording growth patterns of MTB. When compared with current phenotypic methods for TB detection and susceptibility testing, MODS has proven to be more sensitive, faster, and cheaper, and is performed directly on sputum samples. The incorporation of TB drugs into the culture medium allows for the determination of antimicrobial sensitivity to rifampin and isoniazid. MODS uses liquid culture media in 24-well plates inoculated with decontaminated sputum, incubated at 37°C. Observations are made every other day in a light inverted microscope to detect cording colonies as evidence of TB positivity. TB colonies go through three visually distinct stages of growth (Figure 1). In average, in the first 5–6 days, small ‘comma’-shaped objects start to appear. Most characteristically, after 7 to 10 days in MODS culture, clusters of bacteria with a cording pattern appear. After 10 days, the cording colonies begin to aggregate to form clusters or conglomerates. The cording pattern of colonies at 7–10 days is specific for MTB, and is the basis of MODS diagnosis [7], [8]. Laboratory experts in MODS identify five discriminating visual features: a distinct length and thickness, a distinct refraction of light showing a higher brightness along the central axis of the cord, unique extremities with sharp-appearing ends, and an overall sinusoidal form [7]. MODS diagnosis is currently performed based on the manual human observation of these qualitative characteristics. However, each of these characteristics can be mathematically defined, and thus lend themselves to automated analysis.

### Samples and image digitalization

765 digital photos from 7–10 days MODS cultures were analyzed. Of these, 320 were from cultures positive for MTB, 109 were from cultures growing non-tuberculous mycobacteria (NTM) (42 *Mycobacterium kansasii*, 31 *Mycobacterium avium*, 36 *Mycobacterium chelonae*) and 336 were from cultures negative for mycobacteria. From these photos, a total of 5832 objects were selected (2445 MTB cords, 307 *M. kansasii*, 320 *M. avium*, and 312 *M. chelonae,* and 2448 objects from cultures negative for mycobacteria). The MTB objects and the non-tuberculous mycobacteria objects were identified and selected from all the photos, by two MODS experts. Every positive object identified was included. The non-mycobacteria objects obtained from the cultures negative for mycobacteria corresponded to a random sample of all the objects identified in the corresponding photos. Objects and photos were classified and randomly matched into a training set (2446 objects, 328 photos) and testing set (2447 objects, 328 photos). All samples were provided anonymously.

**Figure 2:**
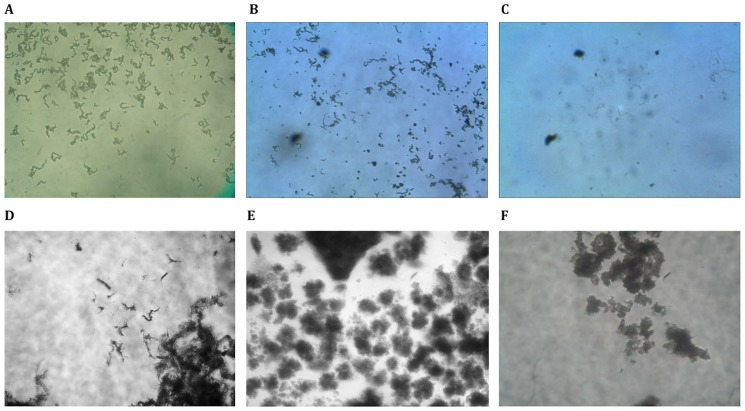
Digital photos of MODS cultures. Corresponding to: (A, B) *Mycobacterium tuberculosis*; (C) Objects from sediment and detritus of a mycobacterium negative sample; (D) *Mycobacterium kansasii*; (E) *Mycobacterium avium*; (F) *Mycobacterium chelonae.*

MODS culture photos were obtained using an inverted light microscope (NIKON Eclipse TS100-F with an infinity correction optical system) with 100X magnification (10X objective and 10X eyepiece) and a CCD (charge-coupled device) digital camera (Olympus C-3030) with 3.34 Megapixel resolution attached to the photo port. Images were captured with the digital camera set to its maximum optical zoom (3X). Typical images of the cording colonies of MTB and the mycobacterial non-cording colonies of NTM are shown in Figure 2.

### Image processing

**Figure 3:**
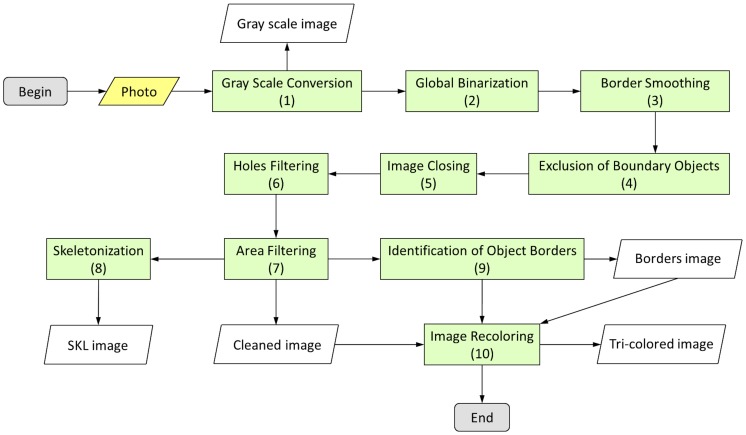
Flowchart of the image processing algorithm. The original photo (in yellow), is processed by the ‘image processing algorithm’ (in green). The numbers inside the green boxes correspond to the steps described in the manuscript. Five images are obtained: Gray scale image, SKL image, Borders image, Cleaned image and finally the Tri-colored image, which is built using the Cleaned image and Borders image.

To identify objects in a digital image of a MODS culture, for further analysis and pattern recognition, a 10-steps image processing was applied sequentially. A flowchart of the image processing is shown in Figure 3:

**Conversion to gray scale and preliminary processing.** To reduce the variability of color and intensity due to the multiple orientations of the objects (in particular the mycobacteria cords) with reference to the light source, all images were converted to a gray-scale according to the standard NTSC values. The 256 gray-scale levels from the digital images were transformed to a proportional scale between 0 and 1. The original red-green-blue (RGB) photographs were transformed by the NTSC standard, which is based on an optimal human perception [14]:

I  =  0.2989*Red + 0.5870*Green + 0.1140*Blue

The image obtained after this process is called “Gray scale image”.

**Global binarization.** To filter the objects from the background, images were binarized by transforming them from gray scale into black and white. The binarization method used the Otsu algorithm [15] which calculates a global threshold value based on all the pixels of the image. Each pixel was transformed into either black or white.

**Border smoothing.** To enhance the objects contours, the edges of digital objects were softened by the application of a third-order median filter [16], [17]. With this treatment, pixels that caused irregularities (‘noise’ pixels) in the continuity of the borders were removed by maintaining no more than four "noise" pixels in the 8-pixel-neighborhood (the eight immediate neighbor pixels of each pixel).

**Exclusion of boundary objects.** The objects that crossed the external border of the image were excluded to prevent inaccuracy in the analysis of pattern recognition. These objects were selected by identifying pixels that intersected with the external boundaries of the image.

**Image closing.** To further reduce the noise along the borders of the objects, their contours were modified and smoothed by dilatation followed by erosion morphological operators [18].

**Holes filtering.** Removal of the white regions inside the black binarized objects (called white sub-objects), was made by labeling the sub-objects followed by a black recoloring.

**Area filtering.** Used as a first filter, objects exhibiting extreme values of area were removed. The 95% confidence interval (CI) of the area from 1200 objects corresponding to MTB cords was first estimated. Objects with an area not included within the 95% CI were eliminated. The image obtained after this process is called “cleaned image”.

**Skeletonization.** A forestation-based algorithm [19], [20] was used to shrink the objects, obtaining an approximation of its overall shape and retaining its major topological features. This digital path was called the “skeleton”. The image obtained after this process is called “SKL image”.

**Identification of object borders.** In order to identify the border of the objects, a Sobel's gradient operator was applied. This operator uses vertical and horizontal masks to extract all the pixels in the border of an object. The image obtained after this process is called “Borders image”.

**Figure 4:**
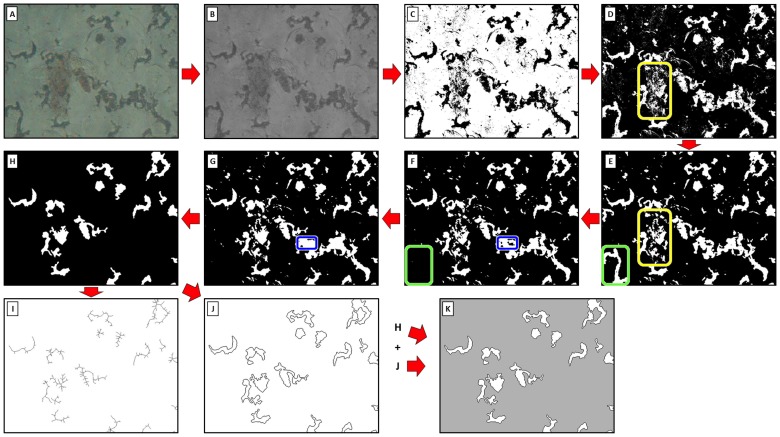
Steps of image processing of a MODS-culture photo. (A) Original photo. (B) Conversion to gray scale, obtaining the ‘Gray scale image’. (C) Global binarization, transforming all the pixels in black (0) or white (1). (D) Black-white inversion. (E) Border smoothing, to reduce the ‘noise’. The yellow rectangle in figures D (before) and E (after) shows the changes produced by this process. (F) Exclusion of boundary objects, deleting the objects in the border of the photo. The green rectangle in figures E (before) and F (after) shows the changes produced by this process. (G) Holes Filtering, removing black objects inside white objects. The blue rectangle in figures F (before) and G (after) shows the changes produced by this process. (H) Area filtering, dropping the outsider values in the distribution of area values, obtaining the ‘Cleaned image’. (I) Skeletonization, obtaining the ‘SKL values’. (J) Identification of object borders, obtaining the ‘Borders image’. (K) Image recoloring: The black pixels in figure H are converted to gray, and this picture is superposed by the figure J, giving the ‘Tri-colored image’. Figures (B), (I) and (K) are used for the features extraction process.

**Image recoloring.** To facilitate further visual analysis, a tri-color system was adopted by using a re-coloring script. The background was colored gray; the border was colored black, and the interior of the object white. The image obtained after this process is called “Tri-colored image”. Figure 4 shows a detailed example of the image treatment and the selection of objects in a digital image of a MODS culture.

### Features extraction and pattern recognition

**Figure 5:**
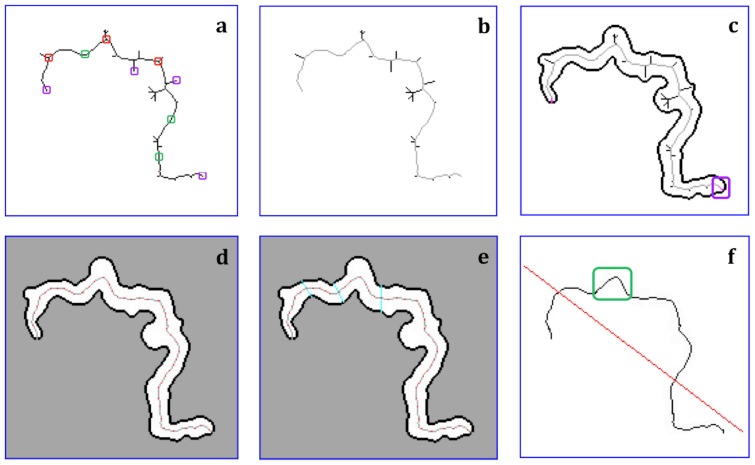
Morphological characteristics of a *Mycobacterium tuberculosis* cord from a TB-positive MODS culture. The process performed to obtain the main features of a *M. tuberculosis* cord includes the following steps: (A) Skeleton of the object, (in purple an example of end-points, in red branch-points, and in green inner-points); (B) Identification of the trunk (gray) and its branches (bold black); (C) Extension of the skeleton. The extension of the trunk (in red) from each end-point of the trunk to its nearest tip of the object (enclosed in purple), and the border of the object (bold black); (D) The extended trunk of the object divided by equally-spaced segments (in red, on the gray skeleton); (E) Transversal division of the object by segments (in cyan) perpendicular to and equally spaced along the extended trunk; (F) The “linearization” of the extended trunk (red line) and waves recognition (one wave is enclosed in green as example).

The length, shape, thickness, brightness-distribution, and circularity of each object were estimated among other parameters detailed below:

**Length of the object.** The length of the object was calculated from characteristics of its skeleton. The skeleton of an object is composed by its trunk and branches. To identify the parts of the skeleton, each pixel was classified with respect to its 8-pixel neighborhood. Three critical types of points along the skeleton were identified allowing differentiating the trunk and its branches: end-points, branch-points, and inner-points (Figure 5a). End-points on the skeleton were identified as pixels in the SKL image with only one black pixel in its neighborhood. Starting at one end-point, pixels with at least three black pixels in their neighborhood (branch-points) were identified. Pixels with only two black pixels in their neighborhood were classified as inner-points, which are part of the trunk or the branches. To distinguish the trunk and branches, the skeleton was transformed into a completely weighted undirected graph. In this, a “vertex” is defined as an end-point or branch-point. In the graph, each vertex is linked with its “neighbor vertex” by following the digital path from one vertex to the other vertex along the skeleton using an “arc”. This was labeled with the digital length between these vertices. The trunk was defined as the longest path between two end-points along the skeleton, and its length is the diameter of the weighted undirected graph. A branch was defined as the shortest path between one branch-point in the skeleton and one end-point outside the trunk (Figure 5b).

To extract the border of the object from the gray background, a vertical line was drawn upward from a skeleton end-point. Along this line, the first black pixel with at least one neighbor of the background color was chosen as the first border point. From this point, clockwise iterations were performed to find the path of black pixels, which form a continuous and closed-edge loop around the object.

To extend the trunk until the border of the object, the trunk was linked from each end-point to its nearest “skeleton-end”. A skeleton-end is a tip of the object, and was located from an end-point, by the following process:

From an end-point, we draw a transversal line (perpendicular to the trunk) until intersects the border and dividing it in two fragments. We select the fragment with the shortest border and select the point that divided the border of this fragment in equal lengths. From this point, we draw 16 equiangular radial transversal lines (randomly oriented). The segments that connected the original point with the intersection with the border were defined, and we selected the segment with the minimal length, provided that it was completely included in the object. Using this segment, we repeated all the process described above. This process stopped when the segment with minimal length obtained from a point ‘i’ is greater than the length of the segment obtained from the point ‘i-1’.

The extended trunk plus all the branches define the ‘complete skeleton’. The length of the object was defined as the number of pixels included in the extended trunk (Figure 5c). For each object, we calculated the number of tips of the complete skeleton as an indicator of the smoothness of the digitalized object.

**Thickness of the object.** Points every three pixels were selected in the extended trunk (Figure 5d). From these points, transversal segments were drawn perpendicularly to the extended trunk, until intersects the border of the object (Figure 5e). The length of each transversal segment in an object was calculated, and from them some parameters were defined: “width” (mean of these lengths), “SD-width” (standard deviation of these lengths) and “max-width” (maximum value of these lengths). With these, features, three new parameters were defined, after normalizing their values by the length of the extended trunk: rel-width, rel-SD-width, and rel-max-width respectively.

**Brightness distribution of the object.** The spatial variation of the brightness in the object as a result of light refraction was measured within each transversal segment. For each pixel in a segment, the difference of the pixel brightness in gray scale and the minimum brightness in this segment was calculated. If this difference was above certain cutoff of expected variation, the pixel was selected. The increase in the percentage of brightness, compared with the mean brightness of the photograph, was analyzed using different cutoffs: 8%, 10%, 12%, 15%, 18%, 20% and 22% and were recorded in parameters with suffix “refr” (“refr8” for cutoff 8%, as example). The number of pixels in a segment that met this condition was normalized by the total number of pixels included in this segment. After considering all the segments within each object, the maximum (max-Nrefr), minimum (min-Nrefr), average (mean-Nrefr) and standard deviation (SD-Nrefr) of the normalized values were calculated for each object.

**Circularity of the object.** The circularity parameter was calculated for each object as 4π times its area divided by the square of its perimeter. The perimeter was calculated as the length of the border of the object, which was measured as the number of pixels included in it. In a perfect circle, this parameter will be equal to 1. A higher value of this parameter represents a lower circularity, as occurs in a cording-shaped object of a TB MODS culture.

**Shape of the object.** The shape of an object was estimated as the shape of its skeleton. Three different methods were used to measure the shape:

Linear fitting: The linearity of the skeleton was estimated by constructing the regression line fitting the pixels contained in it (Figure 5f, red line). Two parameters were calculated: The mean (parameter “mean-for”) and the standard deviation (parameter “SD-for”) of the squared Euclidean distance between the pixels of the extended trunk and the fitted line.

Curvature: The discrete points of the extended trunk were fitted into a continuous non-linear function using a Gaussian fit. The curvature value of each point was estimated by the Fourier’s Fast transformation (FFT).

**Figure 6:**
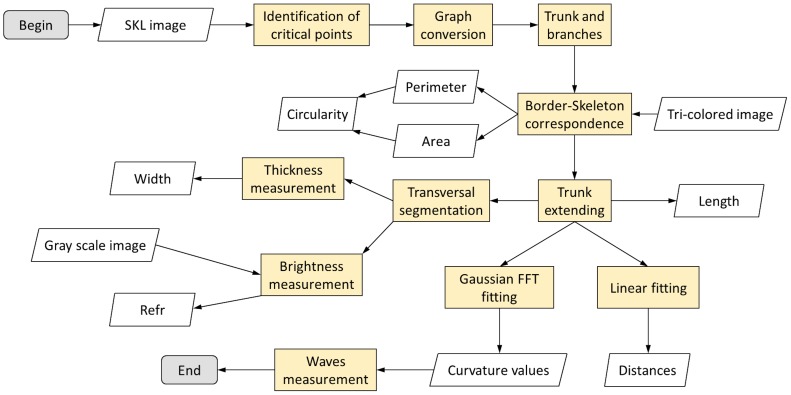
Flowchart of the features extraction algorithm. The SKL image, the Tri-colored image and the Gray scale image are used as input in different steps of the process (in orange) to extract the features. From this process 53 features are obtained for the statistical analysis.

Wave analysis: Along the extended trunk, and using the curvature values obtained, a wave was defined as a set of pixels between two points of minimum curvature (≤0.2) “A” and “B”, which undergo a change of curvature (>0.4) when moving from A to B (Figure 5f, in green). The degree of sinuosity was estimated as the number of waves in the extended trunk and the size of each wave (in pixels). The number of waves, the size and curvature of each wave (defined as the maximum curvature value in the wave) was estimated. A complete flowchart of the process of features extraction is shown in Figure 6.

### Model building and statistical analysis

**Object-models.** A training set (N = 2446 objects) and a testing set (N = 2447 objects) were analyzed to build and evaluate the best object-model to predict TB-positive objects. A total of 2446 objects (1222 TB-positive and 1224 TB-negative) were analyzed. Each digital object was classified according to the features described above. The variability of each feature was expressed as a distribution, and a comparison was performed between the groups of TB-positive and TB-negative in a univariate analysis. A T-test was used to compare the means and a simple logistic regression was used to model the binary response variable (presence or absence of TB) having each feature as the predictor covariate. Features that were significantly associated with TB classification in the univariate analysis were considered for a multiple regression analysis. Features that were highly correlated (Pearson or Spearman rho over 0.75) were dropped. The feature with the highest odds ratio in the univariate analysis was selected and included in the multiple regression analysis and the correlated variable was dropped.

The probability of a positive TB cord was modeled in a multiple logistic regression. A saturated model including all the covariates was first calculated. Covariates with the least significance were removed one at a time, while nested models were compared with the likelihood ratio test. The best object-model with the fewest covariates was obtained. Alternatively, we built a best multiple object-model with the step-wise method by adding one covariate at a time starting with the most significant. Nested models were compared with the likelihood ratio test. In every case, outliers were detected using the test of Hadi, and interactions between features were tested. We demonstrated that both approaches converged into a best multiple model.

The sensitivity and specificity of the best object-model was calculated. Positive objects were used to estimate the sensitivity, and negative objects were used to estimate specificity. Several cutoffs for the probability of TB positive were evaluated, and the best object-model was chosen as the one that maximized the Youden’s J-index (J = sensitivity+specificity-1) [21]. The area under the Receiving-Operator-Curve (ROC), estimated the accuracy of the model as a diagnostic test.

Alternatively the best object-model was used to classify the 2447 objects of the testing set (1223 TB-positive and 1224 TB-negative objects). A sensitivity and specificity was estimated after classifying all objects on this set.

Four object-models were constructed for different sets of objects selected by a skilled technician: constructed from ‘TB cords’ (N = 1100) versus ‘non-cording-objects’ (N = 1078) from TB-positive cultures (object-model-1); constructed from ‘TB cords’ (N = 1222) from TB-positive cultures versus ‘TB-negative objects’ (‘sediment/detritus’)(N = 1224) from TB-negative cultures (object-model-2); constructed from ‘TB cords’ (N = 1228) from TB-positive cultures versus ‘sediment/detritus’ (N = 650) from TB-negative cultures and ‘non-cording-objects’ (N = 667) from TB-positive cultures (object-model-3); constructed from ‘TB cords’ (N = 1228) from TB-positive cultures versus ‘sediment/detritus’ (N = 309) from TB-negative cultures, ‘non-cording-objects’ (N = 309) from TB-positive cultures, and NTM (*M. kansasii* (N = 307), *M. avium* (N = 320), *M. chelonae* (N = 312)) (object-model-4).

**Photo-model.** A total of 652 photos (330 TB-positive and 322 TB-negative) from MODS cultures were analyzed to build the best photo-model. Each digital photo was assigned a set of covariates, which were quantified into a numerical variable for analysis.

From each photo, objects were selected, and the four object-models were applied on all of them. For each photo and for each object-model the eight best objects with the highest probability-score were selected. All these probability scores were used as predictors to classify a TB-positive photo. Additionally, the mean object-probability score was calculated for the best-two, best-three, best-four, best-five, best-six, best-seven, and best-eight objects for each photo and for each object-model.

For each photo the total number of objects identified during the image processing step was obtained, along with the mean and standard deviation of the complete photo brightness. The total number of TB-positive objects (cords) was calculated for each photo.

All these covariates were used as predictors in a multiple logistic regression to model the probability of being a TB-positive or TB-negative photo. Features that were not significant in a univariate analysis, or were highly correlated (over 0.75), were not included. Similarly as in the construction of the best object-model, the best photo-model was obtained by selecting the best multiple logistic regression in a step-forward approximation (adding covariates starting from the most significant) and a step-backward approximation (removing the least significant covariates from the saturated model). In all cases the likelihood ratio test was used to compare nested models. The two approaches converged into a best photo-model.

The sensitivity and specificity of the best photo-model was calculated after modeling the probability of being a TB-positive or TB-negative photo. The best photo-model was chosen as the one that maximized the Youden’s index. The area under the Receiving-Operator-Curve (ROC) determined the accuracy of the best photo-model as a diagnostic test.

A dictionary of the main variables tested is found in Table 1. All the statistical analyses were performed with a 5% significance level using the software Stata 11.

**Table 1:** Dictionary of variables.

Name of variable	Description
Width	Mean width of the object
Length	Length of the object
rel_width	Mean width of the object normalized by the length of the main skeleton of the object
SD_width	Standard deviation of the width of the object
Mean-Nrefr15	Average of the number of pixels along the transversal segment to the skeleton that had brightness 15% higher than the average bright of the photograph normalized by the total number of pixels of the particular transversal segment.
bimedia	Mean of the brightness values of the pixels in all the transversal segments of an object
Num-tips	Number of tips of the complete skeleton.
Mean-for	Mean of the squared Euclidean distance between the pixels of the extended trunk and the fitted line.
SD-for	Standard deviation of the squared Euclidean distance between the pixels of the extended trunk and the fitted line.
perimeter/length	Ratio perimeter:length of the object.
max-width	Maximum width of an object.
SD-photo	Standard deviation of the brightness of all the pixels within a photograph.
Num-obj	Total number of valid objects in the photograph
numposi_object-model-2.1	Number of positive objects detected in a photograph according to the statistical model object-model-2.1 (model under the database of positive cords vs. detritus).
numposi_object-model-2.2	Number of positive objects detected in a photograph according to the statistical model object-model-2.2 (model under the database of positive cords vs. detritus).
Mean-2.1_6	Average of the top six highest probabilities of objects from the analyzed photograph.

## Results

The best object-model to classify cords was the object-model 2, considering the eight features shown in Table 2. Object-model 2 showed the best biological significance because it included the features observed by the TB experts.

**Table 2:** Univariate analysis of the main features relevant to the prediction of TB positive objects.

Morphological Features	Features (unit)	Mean	Standard Deviation	Minimum value	Maximum value
Width of object	rel_width (pix.)	11.85	3.48	3.10	39.61
	SD_width (pix.)	4.16	1.70	1.33	26.79
Ilumination of object	Mean-Nrefr15 (prop.)	74.67	3.77	59.27	84.51
	bimedia (brightness units)	51.64	2.61	43.94	62.67
Form and length of object	Num-tips (non-dimensional)	8.52	4.31	3	28
	perimeter/length(prop.)	2.23	0.15	1.97	3.84
	max-width (pix.)	23.56	6.28	11.70	79.19
	SD-for (pix.)	8.54	4.36	0.99	35.85

(pix.): pixels; (prop.): proportion. The statistics shown were estimated from 1223 positive cords.

**Figure 7:**
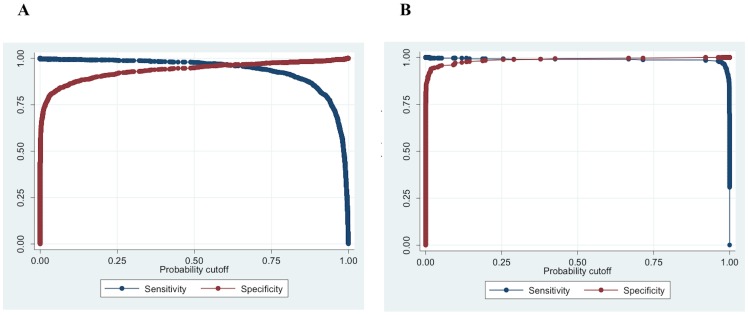
Sensitivity and specificity of the best model to diagnose *Mycobacterium tuberculosis* from a MODS culture digital image. (A) Sensitivity and specificity for different probability cutoffs for the best object-model to classify *M. tuberculosis* cords; (B) Sensitivity and specificity for different probability cutoffs for the best photo-model to classify *M. tuberculosis* positive MODS culture images.

This model was able to identify MTB objects with 96.89% sensitivity and 96.32% specificity in the testing set. The area under the ROC curve was 0.989, and the Youden’s index was 0.93. The variability of the outcome explained by the model was 82% (pseudo-R^2^). The combinations of sensitivity/specificity for the best object-model are shown in Figure 7A. At the object-level, specificity in discrimination of MTB cords from *M. avium* objects was 97.06%, 99.14% for *M. chelonae*, and 93.75% for *M. kansasii*.

The univariate and multiple analyses and the significant covariates involved in the logistic regression of the best photo-model are summarized in Table 3.

**Table 3:** Relevance of features from the best photo model in a univariate and multiple variable logistic regression.

Model	Overall R^2^	Features	Odds Ratio (R^2^, P-value): Simple logistic regression	Odds Ratio (P-value): Multiple logistic regression
Photo Model	0.98	SD-photo	1.21 (0.29, <0.001)	0.73 (0.006)
		Num-obj	1.01 (0.01, 0.011)	0.74 (0.001)
		numposi_object-model-2.1	1.68 (0.58, <0.001)	2.72 (0.003)
		numposi_object-model-2.2	1.25 (0.37, <0.001)	1.84 (<0.001)
		Mean-2.1_6	1.70x10^5^ (0.61, <0.001)	1.18x10^9^ (<0.001)

The best photo-model was able to identify MTB photos of MODS cultures with 99.1% sensitivity and 99.7% specificity in the testing set in 15 seconds in average per photo in a P4 2GHz CPU. The area under the ROC curve was 0.999, and the Youden’s index was 0.99. The variability of the outcome explained by the model was 98.0% (pseudo-R^2^). The combinations of sensitivity/specificity for the best photo-model are shown in Figure 7B.

The features included in the best photo-model were the variability of brightness of the photo, number of total objects in the photo, number of positive objects identified according to the object-model-2 (variant 1), number of positive objects identified according to the object-model-2 (variant 2), and the average score of the six best objects identified by object-model-2. The univariate and multiple analyses and the significant covariates involved in the logistic regression of the best object-models variants are summarized in Table 4.

**Table 4:** Relevance of features from the best two variants object models, in a univariate and multiple variable logistic regression.

Model	Overall R^2^	Features	Odds Ratio (R^2^, P-value): Simple logistic regression	Odds Ratio (P-value): Multiple logistic regression
Object model 2.1	0.82	rel_width	0.74 (0.37, <0.001)	0.69 (<0.001)
		SD_width	0.57 (0.28, <0.001)	0.41 (<0.001)
		Mean-Nrefr15	1.17 (0.21, <0.001)	1.25 (<0.001)
		Bimedia	0.81 (0.15, <0.001)	0.78 (<0.001)
		Num-tips	0.93 (0.04, <0.001)	0.78 (<0.001)
		SD-for	1.14 (0.06, <0.001)	1.10 ( 0.004)
		perimeter/length	0.00 (0.40, <0.001)	0.01 (<0.001)
		max-width	0.91 (0.14, <0.001)	1.33 (<0.001)
Object model 2.2	0.84	rel_width	0.74 (0.37, <0.001)	0.66 (<0.001)
		SD_width	0.57 (0.28, <0.001)	0.65 (<0.001)
		Mean-Nrefr15	1.17 (0.21, <0.001)	1.39 (<0.001)
		Bimedia	0.81 (0.15, <0.001)	0.69 (<0.001)
		Num-tips	0.93 (0.04, <0.001)	0.71 (<0.001)
		SD-for	1.14 (0.06, <0.001)	1.08 (0.033)
		Length	1.01 (0.11, <0.001)	1.02 (0.022)

At the photo-level, the cross-reactions of the algorithm with other mycobacteria digital microscopic photos were null. We analyzed 41 *M. Kansasii* samples, 43 *M. chelonae*, 30 *M. avium* and 72 *M. bovis*. No false positive for TB were observed.

## Discussion

Just as humans can be trained to recognize characteristic MTB growth in MODS, we hypothesized that computerized image analysis algorithms could be developed to distinguish positive from non-positive MODS cultures. This study reports for the first time a computer algorithm to diagnose tuberculosis, and by virtue of identifiable growth in the presence of drug, multi-drug resistance by analyzing digital images of a MODS culture. This algorithm is based on the recognition of morphological characteristics from objects of a digital image captured with an inverted microscope, and is able to correctly recognize MTB cords and differentiate NTM with a high sensitivity and specificity.

In the image processing and the pattern recognition components described in this study, we have included ad-hoc quantification of specific features of the objects in order to resemble the ´human proceduré followed to classify a TB cord. The use of multiple logistic regression for classification allowed us to sense the behavior of each predictor (both their adjusted and unadjusted effects), their potential interactions and confounding. The most important features in the recognition of a TB positive MODS culture were geometrical and illumination type characteristics. These highly specific features of TB cording colonies appearing between 7–10 days of culture are the basis of MODS. This study confirms that the predictors of the best object-model that correctly classified MTB cords, were the same features considered in the standard manual-visual inspection. These include the length, width, shape, and the presence of a refraction pattern associated with a higher brightness in the axis of the cord. Interestingly, there were other parameters that appeared to be significantly associated with a positive culture that had not been previously recognized or consciously used as a discriminatory characteristic. These features have likely never been considered before because human visual recognition is a complex process not usually broken down into its constituent elements.

The microbiological quality of the MODS culture, and therefore the integrity of the objects in the digital photo are important and affect the accuracy of both a manual and an automatic classification. Sputum samples need to undergo a prior decontamination process in order to kill most of the common commensal organisms that grow in the oral cavity which contaminate the sputum. Inadequate decontamination can result in contamination of the culture, which masks any evidence of TB cords. In these cases the sample needs to be processed again In some situations, an excess of non-specific detritus and sediment-type objects may appear. The algorithm was trained to filter out these objects, but excessive contamination does increase the probability of finding a false positive TB object.

Our algorithm was developed for the identification of individual cords, which represents an intrinsic limitation of the methodology used, due the necessity of excluding overlapping objects for the analysis, reducing the capability of positive cords detection.

Another limitation is the skeleton-based method for features extraction. The generation of medial axis is vulnerable to noise and the smoothness of the medial axis branches is subject to the smoothness of the boundary, which is in turn based on the resolution and quality of the images[22]. We selected and analyzed 1000 random photos, and summit the skeleton obtained to the qualitative opinion of a MODS expert. The overall opinion was that the skeleton showed to be a good representation of the shape of the object, retaining its main characteristics. This is reinforced by the reproducibility of our method and the high levels of specificity and sensitivity obtained by the algorithm in TB cords recognition.

Not only the quality of the microbiological culture is important, but the quality of the microscope and the digitalization system too. The sensitivity and specificity reported in this study correspond to digital images obtained from the microscope and digitalization system described above. Although the algorithm used global binarization, which is sensitive to the brightness distribution, the Nikon microscope used in this study, as well as other good commercial brands with adequate optics, produce images with a uniformly distributed brightness that are correctly processed.

In a recent study, we developed a prototype of a cheap ad-hoc inverted and digital microscope for TB diagnostics with MODS [23]. This cheap and simple system produced images of enough quality that a human technician could correctly classify them. In order to bring down its price, we used as its illumination system a single $1 dichroic lamp. The limitation of this system is that the images are produced with a non-uniformly distributed brightness. The center of the images has more intense brightness. For the human technician this is not a problem, but in this scenario the algorithm looses sensitivity to classify TB cords. To solve this problem, we found that the use of a local adaptive binarization approach using the Niblack algorithm corrects this limitation and is appropriate for images with non-uniformly brightness distribution (data not shown). This change enabled the algorithm to detect TB cords, but required 7 more seconds of computing calculation. This time trade may very well be justified to enable the use of the detection algorithm while keeping the price of an ad-hoc microscope low.

Pattern recognition software like the one presented here could be used in a 100% automatic platform for high throughput TB diagnostics. In a recent study, we developed an automated MODS plate reader to screen each of the 24 wells of a MODS culture plate [24]. The system is able to capture digital photos that cover the complete surface of every well. These images are transmitted to a controlling PC computer, where the pattern recognition algorithm could analyze photos in real time and produce a fully automated reading of a complete MODS culture plate.

In a previous study, we demonstrated that the transmission of MODS digital images using cellphone telephony resulted in a simple way to diagnose TB remotely by a human technician [25]. It is possible that the MODS pattern recognition algorithm be used to remotely diagnose tuberculosis and detect multidrug resistance from transmitted digital images.

The inclusion of the area filter that eliminates objects out of the 95% confidence interval of the area of MTB cords was important in order to speed the calculation. Although the sensitivity is sacrificed with the use of this filter, we believe that 99.1% sensitivity is appropriate for the trade of improving the time of calculation. In addition, a reduction of MTB negative objects to be included in the pattern recognition process increases the specificity, because it is less likely to misclassify a negative object. However, further refinements to the algorithm could be conceived by screening a larger sample of objects, provided that better computing capacities are available.

Although in MTB microbiological laboratories where MODS is performed, the technicians might be qualified in MODS reading, it is important to highlight, that the use of the TB-MODS remote diagnostics is not expected to be mandatory for every sample. It is possible that less experienced technicians may have doubts assessing problematic samples, in which case a remote aid to interpreting a MODS culture would be useful.

Conclusion: For the first time, a MODS pattern recognition algorithm has been developed. Combining geometric and illumination features enables a sensitive and specific identification of MTB and discrimination of MTB from NTMs in MODS cultures. The availability of this algorithm will facilitate MODS reading strengthening the efforts for TB control.
